# Community-Led Total Sanitation and the rate of latrine ownership

**DOI:** 10.1186/s13104-019-4066-x

**Published:** 2019-01-14

**Authors:** Dagim Afework Zeleke, Kassahun Alemu Gelaye, Fantahun Ayenew Mekonnen

**Affiliations:** Department of Epidemiology and Biostatistics, Institute of Public Health, College of Medicine and Health Sciences, University of Gondar, Gondar, Ethiopia

**Keywords:** Latrine ownership, Community-Lead Total Sanitation, Ethiopia

## Abstract

**Objective:**

Lack of sanitation affecting billions of people worldwide is a serious public health problem of Ethiopia. So, we aimed at examining the influence of community interventions on households’ latrine ownership status in Northwest Ethiopia.

**Results:**

The proportion of households owning latrines were 47% (95% CI 42.5, 52.0). Community Lead Total Sanitation practice in the kebele [AOR = 1.78, 95% CI (1.57, 2.03)], health facilities available in the village [AOR = 2.37, 95% CI (2.14, 2.64)], and increased educational attainment of the head of the household were statistically significantly associated with households’ latrine ownership. So, we recommend expansion of community interventions for those who are not yet reached.

## Introduction

Lack of sanitation is a serious health risk, affecting billions of people worldwide, particularly the poor and the disadvantaged. Globally, 2.4 billion people lack improved sanitation facilities, and almost one billion practice open defecation, resulting in 280,000 diarrheal deaths yearly. The magnitude of the problem varies even across developing regions of the world; sub-Saharan Africa, where 50% of the inhabitants practice open defecation, is the most heavily affected region. The situation seems less prevalent in Ethiopia in which 29% of the population were reported to practice open defecation [1].

Open defecation is highly associated with the transmission of diarrheal or gastrointestinal diseases and parasitic infections like hookworm and ascariasis [2].

In Ethiopia, about 60% of the disease burdens are due to poor hygiene and sanitation [3]; people with private and clean latrines are less prone to be affected by poor sanitation related communicable diseases [4–8]. However, the practice is affected by different levels of factors which include, socio-economic, knowledge and attitude, and environmental characteristics in addition [9–11].

The government of Ethiopia has been aggressively working through the Health Extension Program to make every household in the country have a latrine. As a result, according to the Ethiopian Demographic and Health Surveys (EDHS) report of 2011 and 2016, the proportion of households with latrine facilities nationally increased from 55% in 2011 to 61% in 2016 [12, 13]. However, the progress was significantly lower than the stipulated national target of 100% coverage [14]. On the other hand, evidences from small studies conducted in the different places revealed that latrine availability was highly variable across the Ethiopia, as high as 93.5% in Debre Tabor town [15] and as low as 58.4% in the suburbs of Bahir Dar town [11], for instance. This suggests that more research needs to be conducted in specific places with different contexts in the country to provide concerned bodies with evidences necessary for increasing latrine coverage as no study has been conducted to assess the situation in the study area. Therefore, we aimed at evaluating the coverage of latrine and factors contributing to it in Northwest Ethiopia.

## Main text

### Methods

#### Study design and setting

We conducted a community based cross sectional study among households in Dabat district from March to April 2017. The district is located in northwest Ethiopia. It is 821 km northwest of Addis Ababa and is serving as a surveillance site of the University of Gondar since. The district comprises 30 kebeles (the smallest administrative units in the hierarchy of government administration in Ethiopia) with 6 health centers and 35 health posts. It has a total of 41,697 households and 179,295 inhabitants, according to the population projection for 2017.

#### Sampling and size determination

We employed a single population proportion formula for calculating the sample size. We considered a prevalence of 93.5% from a previous study [15], confidence level of 95% and a margin of error 3%. The sample size calculated was 260. As the sampling process was multi stage, we assumed a design effect, and we took a design effect of 1.5. The final sample size obtained after adding 10% non-response was 429.

#### Data collection

We used a pretested structured questionnaire for the data collection. Observation was used to collect data on latrine characteristics and interview for data on variables, which included socio demographic, health facility accessibility and utilization related. The interviewees were heads of households. The questionnaire was originally prepared in English and translated to Amharic (the local language), and responses were then translated to the English language. The thirteen data collectors and two supervisors of the surveillance site collected the data. The data collectors trained for 2 days were deployed with the codes of the households selected randomly from the surveillance site household Registration Book.

#### Data quality assurance

To enhance the quality of the data, we pretested the questionnaire, and accordingly, modifications were made on the questions. We gave training to data collectors and supervisors on the nature of questions, approaching and interviewing participants, and inspecting latrines. The clarity, consistency and accuracy of responses were checked both on the field and in the office. Data were entered by two researchers and cross checked to correct entry errors.

#### Data processing and analysis

We entered the data into epi info version 7 and exported to Stata version 13 statistical package analysis. Descriptive analysis was carried out first to see the distribution population with regard to socio-demographic, geographic, health facility and latrine related characteristics. Means, percentages, graphs, and tables were used accordingly to describe the data. We fitted binary logistic regression model to identify factors associated with latrine ownership. The crude and adjusted odds ratios with their corresponding 95% confidence intervals were computed. Predictors with ≤ 0.05 p-value in the multivariable regression were considered as statistically significant.

#### Ethical consideration

Before starting the study, we obtained ethical clearance from the Institutional Ethics Review Board of the University of Gondar. Permission letters were secured from the different levels of government administration offices. Informed oral consent was obtained from the participants after giving them due information relating to the purpose, benefits, and risks of the study, and the confidentiality of data that personal identifiers of participants were not included in the questionnaire. The respondents were also told that participation in the study was completely voluntary.

### Results

#### Socio demographic characteristics

All (429) of the study households completed the study. Out of the total households, over two-third, 281 (65.5%), were from rural kebeles, and over half, 265 (61.8%), were at least 10 km from the center of the district. Most, 343 (79.9%), of the heads of households were married, and more than half, 237 (55.2%), were not able to read and write. Over half, 238 (55.5%), of the households had at least 5 members, and below one-fourth, 70 (16.3%), had a member with either some level of official authority or membership in the health development army (HAD). Over three-fourths, 340 (79.3%), of the households had school children in the houses. Over half, 239 (55.7%), of the households lived in the highland and the rest in the lowland or semi highland areas of the district. Over half, 232 (54.1%), of the households were in villages where health institutions (health centers or health posts) were available. About two-third, 282 (65.7%), said that there was CLTS carried out in the kebele (Table 1).

**Table 1:** Socio-demographic characteristics of household in Dabat District, Northwest Ethiopia, From March to April, 2017

Variables	Frequency (%)
Residence	
Urban	148 (34.5)
Rural	281 (65.5)
Marital status	
Married	343 (79.9)
Unmarried	6 (1.4)
Widowed	44 (10.3)
Divorced	36 (8.4)
Educational status	
Unable to write and read	237 (55.3)
Able to write and read	96 (22.4)
Primary level	40 (9.3)
Secondary level	39 (9.1)
College and above	17 (3.9)
Household size	
≤ 5	238 (55.5)
> 5	191 (44.5)
Kebele distance from the town	
≥ 10 km from town	164 (38.2)
< 10 km from town	265 (61.8)
Schooling children in the household	
Yes	340 (79.3)
No	89 (20.7)
Official authority or HDA in the kebele	
Yes	70 (16.3)
No	359 (83.7)
Health institution in the kebele	
Yes	232 (54.1)
No	197 (45.9)
CLTS conducted in the kebele	
Yes	282 (65.7)
No	147 (34.3)
Climate	
Lowland	126 (29.4)
Highland	239 (55.7)
Semi highland	64 (14.9)

#### Latrine ownership and related characteristics

This study revealed that 47% (95% CI 42.5, 52.0) of the households in the study area owned latrine. Of all latrines, over two-thirds, 139 (68.5%), had adequate ceilings and walls, and were graded as good. Below one-fourth, 48 (23.7%), of the latrines had no covers or walls, but confirmed as being used by household though they were classified as bad latrines. The least number, 16 (7.8%), which had no ceilings were covered with plastic and considered as fair latrines. The mean duration of latrines in the district was 48 months with a standard deviation of ± 41 months. The mean distance of latrines from houses was 9.4 m with a standard deviation of ± 6.7 m. The main reason for 94 (46.3%) of the households for constructing latrines was the health education given by the health extension workers (HEWs). Of the respondents who did not have latrines, more than half, 126 (55.7%), said the destruction of their previous latrines was the reason for their not having them at the moment (Table 2).

**Table 2:** Ownership of latrine and related characteristics in Dabat District, Northwest Ethiopia, from Match to April, 2017

Variables	Frequency (%)
Had private latrine	
Yes	203 (47.0)
No	206 (53.0)
Latrine feature	
Hole without cover	48 (23.6)
Latrine without enough ceiling and wall	14 (6.9)
Latrine with enough ceiling and wall	139 (68.5)
Latrine covered with plastic	2 (1.0)
Distance of the latrine from home (m)	
< 10	152 (74.9)
≥ 10	51 (25.1)
Duration of latrine (years)	
≤ 7	166 (81.8)
> 7	37 (18.2)
Reason for constructing latrine	
Afraid of penalty	9 (4.4)
Latrine construction campaign	35 (17.3)
Due to HEW supervision	94 (46.3)
Self-motivation	63 (31.1)
Others	2 (0.9)
Reason for not having latrine	
Lack of land	42 (18.6)
Lack of money	20 (8.9)
Lack of awareness	21 (9.3)
Destroyed latrine	126 (55.7)
Other reasons	17 (7.5)

#### Factors associated with latrine ownership

Independent variables like marital and educational statuses, and having official position in the kebele of household heads, income, family size, presence of schooling children, household supervision by health extension workers, CLTS conducted in the kebele, presence of health institution in the kebele, kebele distance from the town and climatic condition were considered in the bi-variable logistic regression analysis. In the final multivariable model however CLTS conducted in the kebele, educational status, presence of health institution in the kebele and climatic conditions were statistically significantly associated with household’s latrine availability. The study revealed that as the level of educational status of the heads of the households increased there appeared increases in the availability of latrines: primary education (AOR = 2.20, 95% CI 11.7, 2.8), secondary education (AOR = 8.90, 95% CI 6.7, 9.7), and college and above (AOR = 10.21, 95% CI 4.9, 21.3). Households in highland climatic areas had 4.29 times higher prevalence of latrines than those who were in lowland climates (AOR = 4.29, 95% CI 3.67, 5.02). The study identified that households that were in kebeles where CLTS was conducted were 1.78 times more likely to have latrines compared to their counter parts, (AOR = 1.78, 95% CI 1.6, 2.0). Households that lived in villages in which health institutions (at least health posts—the lowest level of health care facility) were available were 2.37 times more likely to have latrines compared to those who lived in a villages in which health institutions were not found, (AOR = 2.37, 95% CI 2.14, 2.64) (Table 3).

**Table 3:** Factors associated with coverage of latrine in Dabat District, Northwest Ethiopia, March–April, 2017

Variables	Prevalence of latrine			
	Yes	No	COR (95% CI)	AOR (95% CI)
Educational status*				
Illiterate	91	146	1.00	1.00
Able to write and read	38	58	1.05 (0.9, 1.3)	0.93 (0.8, 1.1)
Primary level	23	17	2.17 (1.9, 2.5)	2.20 (11.7, 2.8)
Secondary level	35	4	14.04 (9.8, 20.2)	8.90 (6.7, 9.7)
College and above	16	1	25.67 (6.5,101.5)	10.21 (4.9, 21.3)
CLTS conducted in the kebele*				
Yes	145	137	1.62 (1.4, 1.9)	1.78 (1.6, 2.0)
No	58	89	1.00	1.00
Climate*				
Lowland	26	100	1.00	1.00
Highland	159	80	7.64 (1.8, 32.2)	4.29 (3.67, 5.02)
Semi highland	18	46	1.51 (0.8, 0.0)	0.99 (0.85, 1.15)
Health institution in the kebele*				
Yes	139	93	3.11 (1.87, 5.16)	2.37 (2.14, 2.64)
No	64	133	1.00	1.00

*AOR* adjusted odd ratio, *COR* crude odd ratio, *CI* confidence interval

* P-value < 0.05 in the multivariable regression

### Discussion

We identified that 47% (95% CI 42.5, 52.0) of the households had latrine. The factors statistically significantly associated with latrine ownership were climatic conditions, CLTS conducted in the kebele, availability of health institution in the kebele, and the educational status of the head of the household.

The coverage of latrines in Dabat district was lower than that reported from studies conducted elsewhere in Ethiopia; suburb of Bahir Dar town (58.4%), Debre Tabor town (93.5%), Kersa district (89.7%) and national coverage (61%) [11, 13, 15, 16]. The differences observed in the finding from suburbs of Bahir Dar town, specifically, may be due to the fact that the suburbs are surrounding the capital of Amhara Regional State in which many non-governmental organizations have been intervening in WASH (Water Sanitation and Hygiene) activities that might have created high public awareness, while the significant difference with the findings in Debre Tabor town and the national survey may obviously be due to the fact that the majority the participants of this study were mainly rural households.

As to the factors responsible for latrine ownership, households found in highland climatic zone were 3.3 times more likely to have latrines compared to households in lowland climatic zones. This is consistent with the finding in Hulet Eju Enessie district, Ethiopia that households in highland climatic zone were more likely to own latrines compared to their counterpart lowlanders [5]. This might be related to variations specifically in the soil and/or temperature in the climatic zones which are believed to affect the construction and durability of latrines [17–19]. Households whose kebeles were subjected to CLTS program were 1.78 times more likely to have latrines compared to those that did not part of the CLTS program. This was also evident in a study conducted in Kersa district where latrine coverage and utilization was high among CLTS implemented-kebeles than those who did not implement [10]. This might happen because CLTS program teaches the community based on evidences, examples and in ways households develop internal motivation on latrine construction and maintenance [20–22]. Households that lived in villages in which health institutions (at least health posts—the lowest level of health care facility) were available were 2.37 times more likely to have latrines compared to those who lived in a villages in which health institutions were not found. This finding corresponds the results reported by a study conducted in the suburb of Bahir Dar town [11] and India [23]. This is because as health facilities become closer to households, there is a possibility for household to get updated health information continuously. This can in part be evident that the prevalence of latrine was significantly increased among urban households as revealed by a study conducted in the suburbs of Bahir Dar and Debre Tabor town [11, 15]. In this study, educational status of heads of the households was found to positively affect the availability of latrines. There is a consistent evidence that educational status positively affects latrine availability [9, 24].

Overall, CLTS conducted in the kebele, proximity of health facility, climatic conditions and educational status were identified to be important predictor variables. Thus, we suggest strengthening and scaling up of community interventions, Community-Lead Total Sanitation program specifically.

## Limitations

Data relating to household income, and use of latrine and hand washing facilities were gathered based on interviewing, which might have resulted inaccurate responses.

## Abbreviations

CLTS: Community Lead Total Sanitation
CSA: Central Statistical Agency
EDHS: Ethiopian Demographic and Health Survey
FMOH: Federal Ministry of Health
HAD: health development arm
HEW: health extension worker
UNICEF: United Nations Children’s Fund
WASH: Water, Sanitation and Hygiene
WHO: World Health Organization
